# A Flare of Hepatitis C Virus-Associated Cryoglobulinemic Vasculitis After COVID-19

**DOI:** 10.7759/cureus.26278

**Published:** 2022-06-24

**Authors:** Kenya Hamazaki, Daichi Umemoto, Tomohiro Asada, Maki Iwatani, Kazuyuki Tsuboi, Koji Oh, Hiroki Konishi

**Affiliations:** 1 General Internal Medicine, Kobe City Medical Center West Hospital, Kobe, JPN; 2 Rheumatology, Kobe City Medical Center West Hospital, Kobe, JPN

**Keywords:** severe acute respiratory syndrome coronavirus 2, coronavirus disease 2019, mixed cryoglobulinemia, mixed cryoglobulinemic vasculitis, hepatitis c virus-associated cryoglobulinemic vasculitis

## Abstract

While undergoing treatment for hepatitis C virus (HCV)-associated cryoglobulinemic vasculitis (CV), a 53-year-old male contracted coronavirus disease 2019 (COVID-19), resulting in a disease flare. Although HCV became negative due to the use of glecaprevir/pibrentasvir, CV remained uncontrolled, and the patient was treated with prednisolone, azathioprine, colchicine, and rituximab. He had not been vaccinated against severe acute respiratory syndrome coronavirus 2 (SARS-CoV-2). He was infected with SARS-CoV-2, likely the omicron variant, and developed a severe illness. However, mechanical ventilation and the administration of remdesivir, dexamethasone, unfractionated heparin, and tocilizumab improved his respiratory failure. Despite improvement in respiratory failure, the patient’s skin lesions and peripheral neuropathy rapidly worsened, followed by the development of intestinal ischemia, which led to death. To the best of our knowledge, this is the first case of acute exacerbation immediately after SARS-CoV-2 infection of HCV-associated CV on immunosuppressive therapy.

## Introduction

Cryoglobulinemic vasculitis (CV) is small-to-medium-vessel vasculitis caused by cryoglobulin-containing immune complexes and is distinguished from cryoglobulinemia, which is asymptomatic. CV is commonly associated with skin lesions, arthralgia, peripheral neuropathy, and renal injury and can lead to life-threatening alveolar hemorrhage, mesenteric ischemia, stroke, and myocardial infarction [1].

Cryoglobulinemia is classified into Type I cryoglobulinemia characterized by monoclonal immunoglobulins and mixed cryoglobulinemia (Types II and III) characterized by monoclonal immunoglobulins. Monoclonal gammopathies, such as multiple myeloma, cause Type I cryoglobulinemia. Mixed cryoglobulinemia is caused by various causes, including chronic viral infections with hepatitis C virus (HCV), hepatitis B virus, and human immunodeficiency virus, autoimmune diseases, such as systemic lupus erythematosus and primary Sjogren’s syndrome, and lymphoproliferative diseases. Chronic HCV infection is the most common cause of mixed cryoglobulinemia, accounting for about 90% of all cases [2].

While coronavirus disease 2019 (COVID-19) is thought to be involved in the development of various autoimmune diseases since the early phases of the pandemic, COVID-19 may cause autoimmune disease flare-ups and worsening of disease activity [3,4]. However, few previous case reports suggest a relationship between CV and COVID-19. Here, we present a case of HCV-associated CV flared up at a time after COVID-19.

## Case presentation

Eleven months before contracting COVID-19, a 53-year-old man with a history of methamphetamine use presented to the hospital with complaints of persistent fever, skin lesions in both lower extremities, and peripheral neuropathy in the extremities. The skin lesions were mainly composed of livedo reticularis, which is relatively atypical for CV, and palpable purpura and skin ulcers were only partially present. The diagnosis of HCV-associated CV was based on the presence of genotype 2b HCV infection, high C-reactive protein, polyclonal hypergammaglobulinemia, hypocomplementemia, positive rheumatoid factor, and positive cryoglobulin. Anti-neutrophil cytoplasmic antibodies and antiphospholipid antibodies were negative, and no other organs were involved. Loss-of-function alleles in the nudix hydrolase 15 gene were negative. In addition to prednisolone 60 mg/day, azathioprine 50 mg/day, and colchicine 1 mg/day, antiviral therapy with glecaprevir (300 mg/day)/pibrentasvir (120 mg/day) was started after methylprednisolone pulse therapy (500 mg/day for three days). Antiviral therapy was completed eight months before COVID-19 when the virologic response was confirmed, and aviremia persisted. Skin lesions, peripheral neuropathy, and inflammatory status relapsed when prednisolone was tapered down to 30 mg/day; hence, rituximab 690 mg (375 mg/m^2^) was administered twice. Rituximab therapy was significantly effective, resolving skin lesions and improving muscle strength to grade 4 in manual muscle testing, although neuralgia remained. Because he remained clinically stable for several months, we were able to resume prednisolone tapering and reduce it to 20 mg/day. However, even though no medication was missed, his skin lesions and inflammatory status worsened again three months before COVID-19, making prednisolone dose reduction impossible. One month before COVID-19, a third dose of rituximab 690 mg (375 mg/m^2^) was administered, and his skin lesions and inflammatory status improved again. Just before the onset of COVID-19, the disease status of his CV was mild as he had no skin lesions and was negative for inflammation.

**Figure 1:**
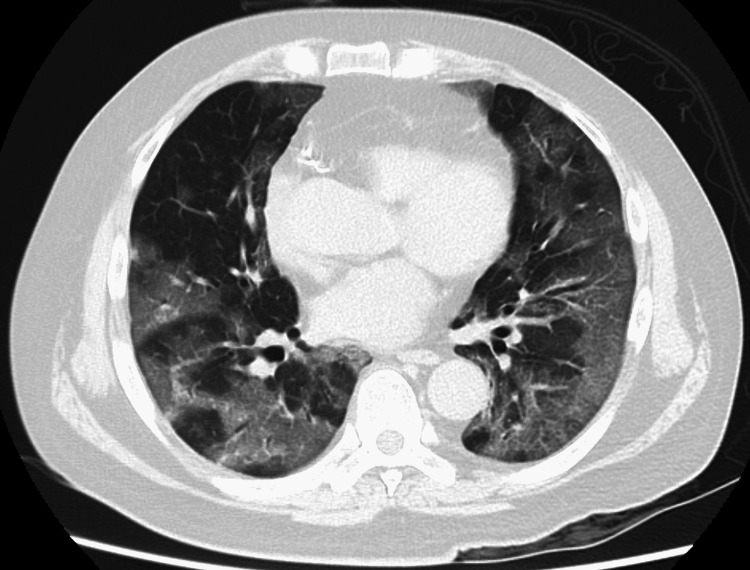
Computed tomography (day 7). A computed tomography scan showed multiple ground-glass opacities in both lungs.

Despite being advised multiple times, he had not received the severe acute respiratory syndrome coronavirus 2 (SARS-CoV-2) vaccine, for reasons including its relatively low efficacy against the omicron variant. He was infected with SARS-CoV-2 at the time when the omicron variant accounted for almost all SARS-CoV-2 cases. From day one, he had a sore throat, fever, and cough, but he did not see a doctor and stayed on his usual medications such as prednisolone and azathioprine. On day 7, he visited the emergency room with severe respiratory distress. Due to severe respiratory failure, he was intubated with mechanical ventilation. A positive reverse transcription-polymerase chain reaction (RT-PCR) test for SARS-CoV-2 led to the diagnosis of acute COVID-19. Physical examination revealed a limited area of livedo reticularis. Laboratory studies were remarkable for C-reactive protein at 17.93 mg/dL and lactate dehydrogenase at 879 U/L. A computed tomography scan showed multiple ground-glass opacities in both lungs (Figure 1). Daily doses of remdesivir 100 mg (200 mg only on the first day), dexamethasone 6.6 mg, piperacillin/tazobactam 18 g, and unfractionated heparin 10,000 U were administered, and a single dose of tocilizumab 600 mg (8 mg/kg) was administered. Respiratory failure and inflammatory status gradually improved, and he was successfully weaned from mechanical ventilation on day 13 and no longer required oxygen supply on day 15. Because the SARS-CoV-2 RTPCR test was repeatedly confirmed negative on both day 14 and day 15, he transferred from the isolation ward to the general ward.

**Figure 2:**
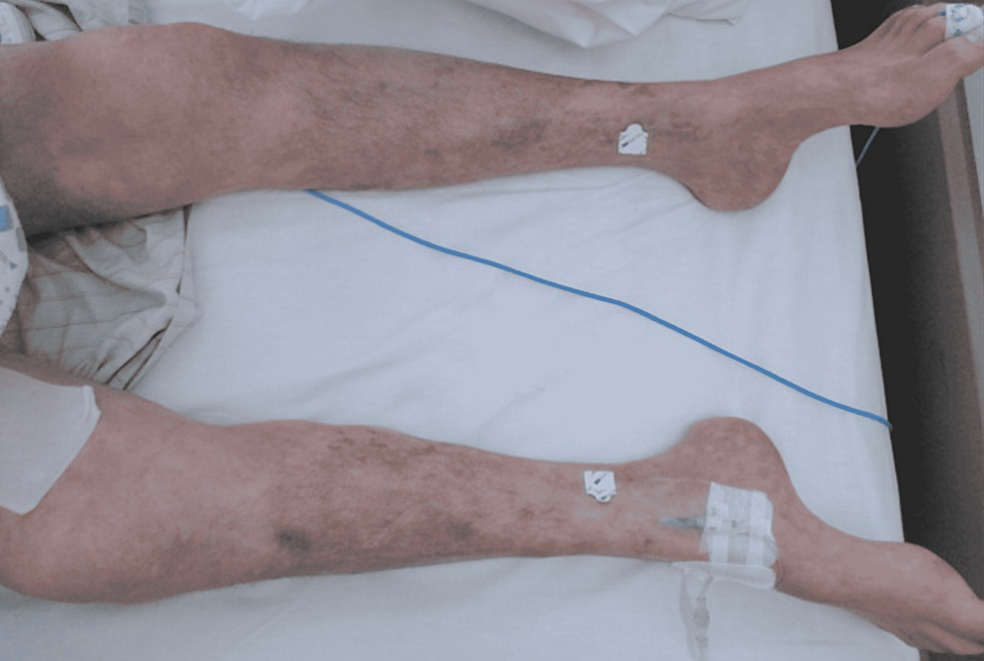
Appearance of both lower extremities (day 15). Skin lesions rapidly spread throughout the extremities.

**Figure 3:**
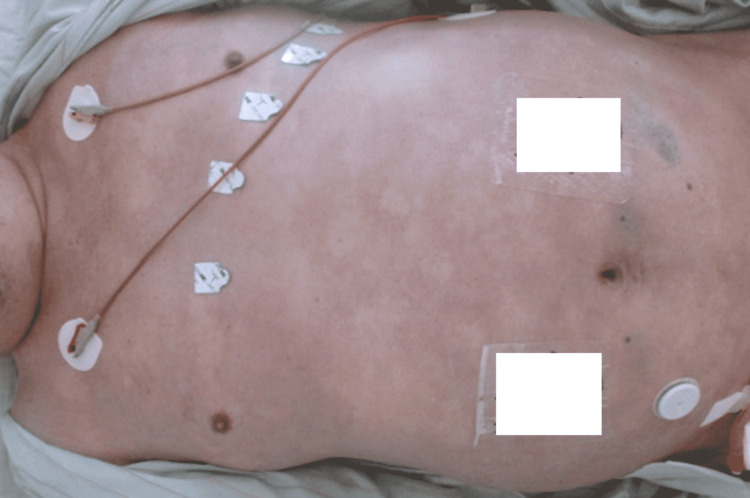
Appearance of the trunk (day 15). Skin lesions also expanded to the whole body trunk.

**Figure 4:**
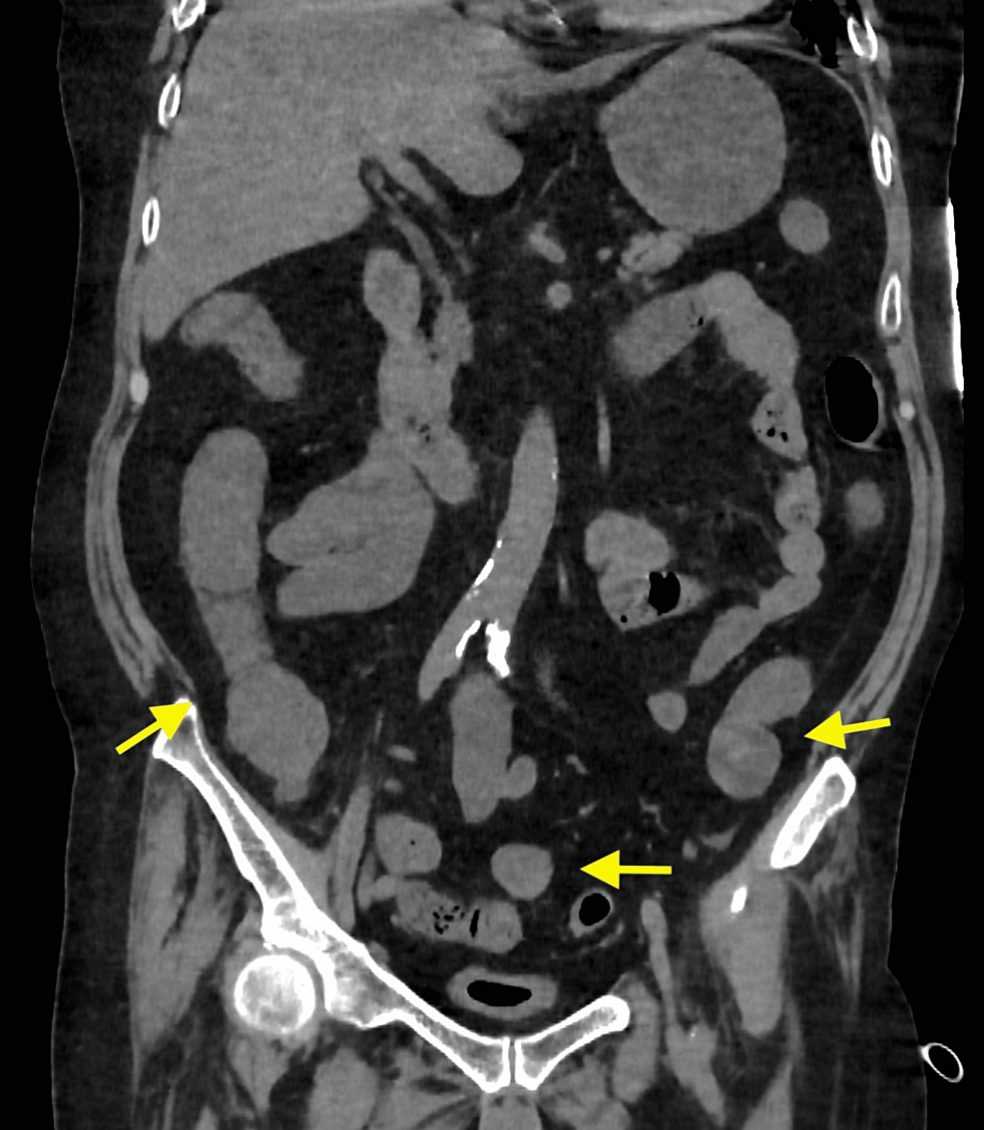
Computed tomography (day 16). A computed tomography scan revealed bowel wall thickening (yellow allows) and fluid retention, suggesting intestinal ischemia. Contrast was preferred, but was not performed due to renal failure.

However, since then, the skin lesions on his extremities, especially livedo reticularis, rapidly expanded to the rest of the body (Figures 2, 3), even though the arteries in the extremities were pulsing strongly and the blood pressure was well maintained. In addition, neuralgia in the extremities was exacerbated, and muscle weakness in the extremities progressed. Myalgia developed in the proximal extremities, and serum creatine phosphokinase (CK) levels increased drastically. CK-MB levels were not elevated, and there were no abnormal findings on electrocardiography or echocardiography. Because it was considered very likely that the CV was acutely exacerbated, a decision was made to perform a plasma exchange; however, severe abdominal pain and hematochezia developed after that, and on day 16, he suffered multiple organ failures (Table 1). A computed tomography scan revealed bowel wall thickening and fluid retention, suggesting intestinal ischemia (Figure 4). Subsequently, his blood pressure dropped, vasopressors and inotropes were given, and mechanical ventilation was restarted. However, he became unresponsive to cardiovasoactive drugs and died without being able to undergo any surgery, such as an intestinal resection.

**Table 1:** Laboratory studies (day 13–16). N/A: not available

Laboratory tests	Reference ranges	Units	Day 13	Day 14	Day 15	Day 16 AM	Day 16 PM
White blood cell	3,900–9,800	/μL	13,000	19,500	22,730	23,080	36,190
Neutrophil	40.0–75.0	%	92.0	N/A	92.5	N/A	95.5
Lymphocyte	18.0-49.0	%	3.0	N/A	1.5	N/A	1.5
Monocyte	2.0–10.0	%	5.0	N/A	2.5	N/A	3.0
Eosinophil	0.0–8.0	%	0.0	N/A	0.0	N/A	0.0
Basophil	0.0–2.0	%	0.0	N/A	0.0	N/A	0.0
Hemoglobin	11.1–15.1	g/dL	15.5	17.0	18.5	18.8	17.2
Hematocrit	33.5–45.1	%	45.7	49.6	56.0	56.0	54.9
Mean corpuscular volume	79–102	fL	92.0	90.0	90.6	90.3	97.9
Platelet count	13.0–37.0	×10^4^/μL	31.1	40.5	45.8	47.4	31.8
Prothrombin time/international normalized ratio	0.90–1.10		N/A	N/A	0.9	N/A	N/A
Activated partial thromboplastin time	23.0–35.0	seconds	N/A	N/A	21.9	N/A	N/A
D-dimer	0.0–1.0	μg/mL	N/A	N/A	4.9	N/A	N/A
Total protein	6.30–8.30	g/dL	5.90	6.20	7.09	6.68	5.21
Albumin	3.80–5.10	g/dL	2.90	3.30	3.62	3.62	2.74
Aspartate aminotransferase	9–35	U/L	98	139	278	272	2,741
Alanine aminotransferase	5–36	U/L	71	97	150	162	2,189
Lactate dehydrogenase	124–222	U/L	754	907	1,134	1,071	3,872
Creatine phosphokinase	56–248	U/L	2,234	3,753	14,402	14,725	11,329
Creatine phosphokinase-MB	0–25	U/L	N/A	N/A	104	104	N/A
Alkaline phosphatase	110–370	U/L	156	162	200	197	225
Gamma-glutamyl transferase	12–70	U/L	N/A	N/A	126	122	207
Urea nitrogen	6–22	mg/dL	46	34	46	57	79
Creatinine	0.47–0.79	mg/dL	0.85	0.64	1.00	1.42	3.63
Sodium	137–144	mEq/L	145	134	133	136	142
Potassium	3.6–4.8	mEq/L	4.5	4.2	5.7	5.7	8.8
Chloride	101–108	mEq/L	109	100	100	103	108
C-reactive protein	0.0–0.5	mg/dL	1.11	0.75	0.47	0.41	0.62

## Discussion

A patient whose HCV-associated CV had been insufficient but reasonably well controlled flared after SARS-CoV-2 infection was described. Although his acute COVID-19 was severe, treatment with tocilizumab, remdesivir, and dexamethasone was successful, and the course of his respiratory failure was relatively uneventful. However, in contrast to the improvement in respiratory failure, skin lesions and peripheral neuropathy quickly worsened, and serum CK levels rapidly rose, followed by intestinal ischemia leading to septic shock and multiple organ failure. Although an autopsy could not be performed, his death was most likely caused by a flare of CV and subsequent intestinal ischemia and sepsis, considering that it had started with a rapid exacerbation of skin lesions and peripheral neuropathy and the absence of any other disease.

CV can rarely cause inflammatory myopathy [5]. While the elevated serum CK levels lacked myocardial damage and were most likely due to dehydration or sepsis, the possibility that they were the result of myopathy due to the CV flare cannot be ruled out as they were accompanied by myalgia in the proximal extremities. Intestinal ischemia is a rare complication of CV but is the primary cause of fatal outcomes of CV. The clinical course was too rapid to recognize the possibility of the CV flare immediately and to proceed with treatment such as plasma exchange in this case. Although we were unable to perform an autopsy and confirm whether the intestinal ischemia was caused by the CV flare, it is possible that we should have performed a plasma exchange or other treatment early on. When a patient with HCV-associated CV was infected with SARS-CoV-2, it may have been necessary to be aware of a CV flare, even if reasonable control had been achieved by antiviral therapy and rituximab therapy.

Viral infection causes autoimmunity through mechanisms such as molecular mimicry, T-cell bystander activation, transient immunosuppression, and overproduction of inflammatory cytokines, also seen in COVID-19 [6]. There is no established theory on whether COVID-19 causes a flare of HCV-associated CV and the details of its mechanism, but there are several interesting references worth considering. A case in which a seropositive patient for HCV had severe COVID-19 and recovered after treatment with remdesivir, steroids, and anticoagulants, but developed purpura of the extremities and trunk and diffuse alveolar hemorrhage and was finally diagnosed as CV was previously reported [7]. This case suggests that COVID-19 may perhaps accelerate the onset of HCV-associated CV, although, of course, it cannot be proven. Two out of three patients with HCV-associated cryoglobulinemia reportedly had persistent inflammatory statuses one month after COVID-19 [8], indicating that SARS-CoV-2 may modify the pathogenesis of HCV-associated cryoglobulinemia through a certain mechanism.

However, a patient with recurrent C3 nephropathy early after renal transplantation and undergoing treatment developed cryoglobulinemia and immune complex-mediated membranoproliferative glomerulonephritis after SARS-CoV-2 infection [9], whereas a patient was reported to have developed cryoglobulinemia and thrombotic thrombocytopenic purpura immediately after COVID-19 [10]. Surprisingly, these patients [9,10] were negative for HCV, suggesting that SARS-CoV-2 may directly induce cryoglobulinemia without HCV infection. In another case report of COVID-19 complicated by cryoglobulinemic glomerulonephritis, interestingly, there was plasma cell dyscrasia in the bone marrow [11]. Further investigation is needed to determine whether and how SARS-CoV-2 infection is involved in the onset and flare of cryoglobulinemia or CV.

Antiviral and rituximab therapy are effective for HCV-associated CV [12,13]. However, it has been demonstrated that rituximab therapy considerably increases the risk of COVID-19 being severe [14]. In this study, there was no increased risk of mechanical ventilation or in-hospital death for the rheumatological, antineoplastic, or antimetabolite therapies, except for rituximab. In addition, the SARS-CoV-2 vaccine is effective in many immunocompromised patients, including our patient on steroids and other immunosuppressive drugs, even though not as much as in the general population, but it is very ineffective in patients receiving rituximab therapy. It has been found that the second dose of vaccination does not significantly enhance the humoral immune response [15] and the additional effect of the third dose is marginal [16]. Furthermore, another study reported that the interval between rituximab administration to SARS-CoV-2 infection did not significantly reduce the severity of COVID-19 [17]. These reports imply that the risk of rituximab therapy is very important in the “With CORONA” era. In other words, clinicians should not only recommend vaccination and instruct on precautions against infection but also reconsider the indication for rituximab therapy. This is also true when a relatively less pathogenic variant such as the omicron is dominant. This is more notable in diseases where alternative therapies are less effective, such as CV and anti-neutrophil cytoplasmic antibody-associated vasculitis than in diseases where several alternative therapies with equivalent efficacy are available, such as rheumatoid arthritis. This problem is further complicated because CV flares have also been reported occasionally after SARS-CoV-2 vaccination [18]. However, because the overall rate of post-vaccination flares observed in CV patients is similar to the rates reported for other autoimmune rheumatic diseases [19], and they resolved spontaneously without endangering patients, we do not see this as a reason to avoid vaccination.

Decisions regarding initiation, continuation, or cessation of rituximab therapy and the timing of vaccination should be made after sufficient discussion with the patients, individually considering the risk of infection with SARS-CoV-2 and their medical conditions. Additionally, patients receiving rituximab therapy should be advised to pay the utmost attention to infection prevention, even if they have been vaccinated.

## Conclusions

We describe a case of HCV-associated CV during immunosuppressive therapy that flared just after infection with SARS-CoV-2. Clinicians should be aware that HCV-associated CV might be exacerbated by COVID-19 even when it is to some extent controlled by antiviral therapy and rituximab therapy; rituximab, which is the most important treatment for HCV-associated CV, increases the risk of more severe COVID-19. The relationship between COVID-19 and the onset or exacerbation of cryoglobulinemia remains unknown and warrants further research.
